# Organizational and Environmental Drivers of Extended Work Hours Among Nursing Staff in Nursing Homes

**DOI:** 10.1093/geroni/igaf122.1496

**Published:** 2025-12-31

**Authors:** Gregory Orewa, Akbar Ghiasi, Justin Lord, Rohit Pradhan

**Affiliations:** The University of Texas at San Antonio, San Antonio, Texas, United States; University of the Incarnate Word, San Antonio, Texas, United States; Louisiana State University Shreveport, Shreveport, Louisiana, United States; Texas State University, San Marcos, Texas, United States

## Abstract

Prior research has shown that extended nursing staff [registered nurses (RNs, licensed practical nurses (LPNs), and certified nursing assistants (CNAs) work hours can lead to more medical errors, declines in care quality, and higher turnover rates. This study examined the organizational and environmental factors associated with extended work hours among nursing staff in nursing homes (NHs). The study utilized multiple datasets such as Care Compare: Five-Star Quality Rating System and Payroll Based Journal, and Data were modeled using multivariable linear regression with year and state-levels fixed effects (2023, n = 42,743). Three separate models were run for for RNs, LPNs, and CNAs. The dependent variable was extended work hours, measured as the percentage of nursing staff exceeding 50 work hours per week, averaged across the year to calculate the annual facility-level rate. The key variable of interest was a categorical variable that captured the intersection of ownership status and chain affiliation: for-profit-chain (FPC), for-profit independent (FPI), not-for-profit chain (NFPC), and not-for-profit independent (NFPI). Results suggested that FPIs and FPCs experienced the highest level of extended work hours, with the largest increases observed for RNs and LPNs. Higher wages correlated positively with extended work hours for RNs and LPNs, but negatively associated for CNAs. NH size was positively associated with extended work hours across all nursing staff. All results were significant at p < 0.001. These findings highlight the need for targeted policy and regulatory interventions to mitigate extended work hours, particularly in for-profit NHs, to improve workforce sustainability and care quality.

